# Psychological Adjustment and Dark Triad Traits in Adolescents Living in Residential Care: A Comparative Study Between Boys and Girls

**DOI:** 10.3390/bs16010037

**Published:** 2025-12-24

**Authors:** Ana Simão, Cristina Nunes

**Affiliations:** University Research Center in Psychology (CUIP), Universidade do Algarve, Campus de Gambelas, 8005-135 Faro, Portugal; csnunes@ualg.pt

**Keywords:** Dark Triad personality, psychological adjustment, residential care, sex differences

## Abstract

Young people in residential care settings tend to present a heightened risk of emotional and behavioral problems. This study intended to explore connections between Dark Triad personality traits and psychological adjustment and to investigate potential sex and age differences in psychological adjustment and the expression of Dark Triad traits. Primary data were collected from a sample of 511 youth (279 girls and 232 boys) aged between 12 and 24 years, living in 46 Portuguese residential care institutions. Self-report questionnaires (Strengths and Difficulties Questionnaire, Short Dark Triad) were used to collect the primary data. Statistical methods were used such as analysis of variance, multivariate analysis of variance, and hierarchical regression analysis. Results showed that boys scored higher in all Dark Triad traits and in behavioral problems. Younger participants scored higher in Machiavellianism and Psychopathy, in emotional and behavioral problems, and in hyperactivity/inattention difficulties. These results could help institutional professionals and social policies assess and delineate individual programs.

## 1. Introduction

Young people typically have their basic needs met within their families; however, some experience violence or neglect, necessitating intervention by child welfare services. In Portugal, more than 5400 youths were placed in residential care institutions in 2023 (Instituto da Segurança Social, 2024). As a protection measure within the Child Protection System, residential care involves placing a child in an institution equipped with adequate facilities and permanent professional staff, responsible for providing safety, care, education, and support for overall development of young people with emotional and behavioral difficulties (Law 142/2015, 2015). Nevertheless, residential care has been associated with higher levels of psychological and behavioral difficulties, as well as lower academic performance and mental health outcomes (Rutter, 2000).

Evidence indicates that youth unable to grow up with their biological parents represent a particularly vulnerable population, showing elevated levels of emotional, behavioral, and social difficulties compared to peers living with their families (Campos et al., 2019; McGuire et al., 2018; Rutter, 2000). Physical and emotional deprivation in substitute care settings has been linked to increased rates of psychosocial, internalizing, and externalizing problems (Magalhães & Camilo, 2023).

### 1.1. Psychological Adjustment of Adolescents in Residential Care

Youth in residential care setting (RCS) frequently experience loss and loneliness, which are associated with psychological difficulties, often reflecting early adverse experiences such as abuse and neglect (Molano et al., 2024).

Psychological adjustment in adolescence refers to the capacity to respond to environmental demands using emotional, cognitive and social resources. Youth in residential care often show greater adjustment difficulties than their peers (Molano et al., 2024), including elevated emotional symptoms, somatic complaints, and behavioral problems (Hayden, 2010; Mayorga-Sierra et al., 2020). Common adjustment concerns in this population include difficulties with impulse control, mood instability, hyperactivity, low self-esteem, and negative self-image (Campos et al., 2019; Molano et al., 2024). These challenges are interrelated and are associated with increased risk of placement instability (Keil & Price, 2006). Adjustment is commonly assessed through internalizing (e.g., anxiety, depression, withdrawal) and externalizing (e.g., aggression) symptoms, which often co-occur (Magalhães & Camilo, 2023; Moreno-Manso et al., 2023), highlighting the importance of considering both domains (Keil & Price, 2006).

Several factors are associated with adolescents’ psychological adjustment in RCS (Simão et al., 2025). Gender differences have been observed, with boys tending to show higher levels of externalizing behaviors such as aggression and rule-breaking, and girls showing higher levels of internalizing problems, including depression and anxiety (Campos et al., 2019; Cotton et al., 2020). Age-related differences have also been reported, although findings are inconsistent. While some studies indicate that internalizing and externalizing problems tend to decrease over time (McWey et al., 2010), others suggest an increase in depressive symptoms during late adolescence. Similarly, research examining the length of stay in residential care has produced mixed findings, with some studies finding associations with better outcomes, others finding no differences, and some reporting higher levels of maladaptive behaviors with longer stays (Costa et al., 2022).

Adolescents may exhibit elevated antisocial traits due to limited responsibilities (e.g., school, family) and social exploration during this period, which can manifest as impulsive or non-normative behaviors. Engagement in prosocial roles has been associated with higher responsibility toward oneself and others. Exposure to adverse experiences may also be associated with differences in behavioral patterns (Bonfá-Araujo et al., 2025). Placement stability appears to be an important factor for psychological adjustment in adolescence, as youth experiencing repeated disruptions after removal from harmful environments show higher levels of psychological and behavioral difficulties (Almas et al., 2020; McGuire et al., 2018). Residential care may pose greater challenges than other out-of-home placements due to high caregiver turnover and limited individual attention (Barone et al., 2016), whereas foster and kinship care tend to provide more stable environments that support emotional and psychological functioning. Given that early relational contexts play a central role in shaping socio-emotional functioning, it becomes essential to examine how care settings may influence key aspects of adolescent development—including personality traits with interpersonal and behavioral implications.

### 1.2. Dark Triad Personality Traits in Adolescents in RCS

Personality refers to biologically influenced traits shaped by environmental factors, reflecting consistent patterns of behavior. Personality traits show plasticity and may vary across contexts and the lifespan (Götz et al., 2020; Muris et al., 2017).

Research has traditionally focused on broad traits such as the Big Five (McCrae & Costa, 1987), which account for general personality variability. However, maladaptive behaviors often arise from interactions among multiple traits, leading to the development of alternative taxonomies to better capture dysfunctional personality patterns within general population samples (Klimstra et al., 2014), including the Dark Triad—Machiavellianism, narcissism, and psychopathy—which capture ethically, morally and socially aversive but subclinical personality traits (Muris et al., 2017). Although considered aversive or undesirable, these traits fall within the normal range of personality functioning and do not, in themselves, imply clinical pathology (Jonason et al., 2015; Jonason & Burtăverde, 2023; Paulhus & Williams, 2002). Therefore, the more precise terms are Machiavellianism, subclinical narcissism, and subclinical psychopathy.

Despite their distinct conceptual foundations, these traits share several core characteristics. All three are associated with a socially malevolent orientation, marked by self-promotion, emotional detachment, manipulation, and, to varying degrees, aggressiveness (Muris et al., 2017; Paulhus & Williams, 2002). Although interrelated, the Dark Triad traits remain distinct constructs. Machiavellianism involves strategic manipulation, emotional apathy, and utilitarian social tactics aimed at exploiting others (Götz et al., 2020; Klimstra et al., 2014). Subclinical narcissism is characterized by grandiosity, dominance, and self-enhancing strategies used to preserve inflated self-views (Klimstra et al., 2014). Subclinical psychopathy is marked by impulsivity, thrill-seeking behavior, and low empathy (Paulhus & Williams, 2002). These traits are associated with maladaptive outcomes, including aggressive, unethical, and antisocial behaviors (Muris et al., 2017; Sijtsema et al., 2019). Research also indicates associations with age, gender, and depressive symptoms (Shen, 2023). Other studies have focused on the potential advantages these personality traits may confer in specific contexts (Diller et al., 2021; Stelmokienė & Vadvilavičius, 2022; Szabó et al., 2024), suggesting that they may serve functional purposes and exhibit adaptive features (Penado Abilleira et al., 2024), specially for males (Jonason et al., 2017).

Interest in dark personality traits among adolescents is growing, yet relatively few studies focus on high-risk populations, such as youth in RCS (Hu & Lan, 2022). Existing research suggests that Dark Triad traits can predict psychological outcomes beyond what is accounted for by the Big Five, highlighting their incremental validity and the need for further investigations (Fernández-del-Río et al., 2020; Koehn et al., 2019) particularly among youth in RCS. Studying these traits in this population is relevant given their higher exposure to early adversity and relational instability, which may be associated with variations in personality expression (Shiner et al., 2017). Investigating Dark Triad traits in adolescents in RCS allows for examinations of associations with behavioral difficulties and psychological adjustment.

Personality can influence cognitive-affective patterns, socioemotional reactions, and behavioral strategies in adapting to current environmental demands (Csathó & Birkás, 2018). This is especially important given that these youth face a variety of challenges and transitions that may intensify the expression of malevolent personality traits. Childhood maltreatment—including emotional abuse, physical aggression, and severe physical punishment—has been associated with socioemotional difficulties and maladaptive personality development (Csathó & Birkás, 2018; Galán et al., 2025). From a theoretical perspective, maladaptive behaviors may arise from environmental influences (Bandura, 1977), socialization processes, genetic factors, peer relationships, or, from an evolutionary standpoint, characteristics that facilitate adaptation and survival in adverse contexts. Such traits may thus be interpreted as adaptive responses to challenging environments that demand specific behavioral strategies (Csathó & Birkás, 2018; Galán et al., 2025; Penado Abilleira et al., 2024).

Evidence shows that Dark Triad traits are observable in youth as young as 11 years (Lau & Marsee, 2013). Older adolescents tend to provide more differentiated self-assessments of these traits compared to younger adolescents, suggesting differences in trait recognition rather than developmental change (Klimstra et al., 2014). Aggression and rule-breaking behaviors also vary across individuals, with environmental factors such as peer affiliation influencing their expression (Tackett et al., 2014). Elevated levels of these behaviors may occur in youth without psychopathology and are considered within normative ranges.

When examining Dark Triad traits, it is important to consider associations with impulsivity, limited self-control, and social skills (Jonason & Tost, 2010), which relate to neurodevelopment, social norms or reduced consideration of future consequences that are characteristic of this developmental period and also linked to some personality traits, such as psychopathy or Machiavellianism (Bonfá-Araujo et al., 2025; Jonason et al., 2017). Developmental psychology posits that personality traits are associated with social adaptiveness across the lifespan, a pattern commonly referred to as the maturity principle of personality development (Schwaba et al., 2022), often showing decreases in socially aversive behaviors with age (Bonfá-Araujo et al., 2025). The maturity principle suggests that with age, individuals acquire greater cognitive resources to regulate their emotions, behaviors, and attitudes, helping them avoid negative social and legal consequences (Schwaba et al., 2022). In this sense, neurobiological, contextual, and experiential factors may shape personality development and contribute to decreases in aversive behaviors and Dark Triad traits (Bonfá-Araujo et al., 2025).

Studies indicate associations between Dark Triad traits and aggression, behavioral dysregulation, and emotional instability in youth (Lau & Marsee, 2013; Tackett et al., 2014). Gender differences have been consistently reported (Götz et al., 2020; Muris et al., 2013, 2017; Pechorro et al., 2022), with boys scoring higher on narcissism and psychopathy, and girls showing higher levels of internalizing problems (Chiorri et al., 2019; Klimstra et al., 2014; Penado Abilleira et al., 2024). Associations have also been found with depressive symptoms (Li et al., 2024; Paknejad & Mazandarani, 2024; Shen, 2023; Wang et al., 2023). Muris et al. (2022) identified psychopathy as the strongest predictor of externalizing problems, with boys scoring higher on both Dark Triad traits and externalizing behaviors, and girls on emotionality. These findings highlight the importance of examining both externalizing and internalizing outcomes when examining socially aversive traits in adolescence. Adolescence is a critical period for depression onset; therefore, exploring this association in adolescence is vital to identify potential risk factors.

This aligns with previous research indicating that Dark Triad traits are significant correlates of adolescents’ psychological adjustment (Klimstra et al., 2014).

Research also suggests that gender differences in Dark Triad traits may be related to differences associated with gender roles (Jonason & Davis, 2018; Penado Abilleira et al., 2024), highlighting the usefulness of understanding the impact of several factors—either internal like age and gender, or external like contextual characteristics (Bonfá-Araujo et al., 2025)—for the development of effective approaches to prevention and intervention.

Regarding age, the literature shows that younger individuals tend to display higher levels of Dark Triad characteristics compared to older adults, potentially due to lower self-control during adolescence (Jonason & Tost, 2010).

Muris et al. (2013) examined all Dark Triad traits in relation to adolescent aggression and delinquency; however, their relatively small sample size highlighted the need for further research on the links between adolescent adjustment and the Dark Triad. Later, Klimstra et al. (2014) found that all Dark Triad traits were associated with direct or indirect aggression in adolescents, reinforcing the importance of examining all three traits to better understand adolescents’ adjustment.

Given the limited research examining the entire Dark Triad within adolescent samples, studying these traits in the context of RCS offers a valuable opportunity to advance the literature. Adolescents in RCS often exhibit social and behavioral difficulties, making this population particularly relevant for such investigation. Notably, in Portugal, youth aged 12 to 17 constitute 50% of those in the child welfare system, with 25% entering due to behavior problems (Instituto da Segurança Social, 2024).

Overall, examining Dark Triad traits in adolescents in RCS provides insight into the relationships between socially aversive personality traits, behavioral difficulties, and psychological adjustment.

### 1.3. Current Study

The primary aim of the present study is to examine the associations between Dark Triad traits and psychological adjustment in adolescents living in residential care. To the best of our knowledge, this is the first study to link self-reports of psychological adjustment in institutionalized youth with their Dark Triad personality traits, while also considering the interrelationships among these three traits. There is substantial evidence supporting the validity and reliability of adolescent self-reports on personality traits. Research indicates that self-reports show reasonable validity and reliability from around age ten, and adolescents’ perceptions of their own personality tend to become more differentiated with age (Klimstra et al., 2014). In the context of this cross-sectional study, examining age-based differences in Dark Triad traits allows for comparison across age groups, without implying developmental change over time.

Given that all three Dark Triad traits fall within the maladaptive personality spectrum and reflect antisocial tendencies, we expected that higher levels of these traits would be associated with elevated internalizing and externalizing problems. Because each Dark Triad trait represents distinct antisocial tendencies, we anticipated that they might show differential associations with internalizing problems (a composite of emotional symptoms and peer difficulties) and externalizing problems (a composite of behavior problems and hyperactivity).

The objectives of this study are twofold: (1) to examine the relationships between Machiavellianism, narcissism, and psychopathy and psychological adjustment in adolescents living in residential care; (2) to investigate potential sex and age group differences in psychological adjustment and the expression of Dark Triad traits.

Based on previous research, we formulated the following hypotheses: (H1) younger participants will exhibit higher scores on the subscales of the Short Dark Triad (SD3) than older participants; (H2) boys will score higher than girls on the SD3 measures; (H3) boys will report higher levels of behavior problems, whereas girls will report higher levels of emotional difficulties; (H4) younger adolescents will display higher levels of adjustment problems than older peers; (H5) Dark Triad traits will be associated with psychological adjustment, with higher levels of these traits linked to poorer adjustment outcomes.

## 2. Materials and Methods

### 2.1. Participants

The sample consisted of 511 adolescents (232 boys, 45%; 279 girls, 55%) aged between 12 and 24 years (M = 15.87; SD = 2.25) residing in 46 Portuguese RCS. Participants were distributed across three age groups: 12–15 years (*n* = 226), 16–18 years (*n* = 227), and 19–24 years (*n* = 58). On average, adolescents had been living in their current institution for 41 months (SD = 43). Participants reported multiple reasons for placement, including neglect and abuse (38%), school absenteeism (33%), financial difficulties (28%), and exposure to domestic violence (26%). More than 40% reported going home on weekends or during school holidays. Additionally, 10% were orphans of at least one parent, and 10% reported no contact with their biological family. While 93% of adolescents had siblings, only 32% were living in the same institution.

The sample size was determined using the GPower 3.1.9.7* (Faul et al., 2007) to ensure sufficient statistical power (≥0.80) for detecting medium effect sizes. Medium effects were defined as *r* = .30 for correlational analyses, d = 0.50 for mean comparisons, f2(V) = 0.15 for MANOVA, and f2 = 0.15 for multiple regression analyses. In cases of uncertainty regarding the expected effect size, conservative estimates (e.g., *r* = .20) were prioritized to reduce the risk of underpowered analyses and Type II errors (Hair et al., 2010). The minimum required sample sized were as follows: 84 participants for Pearson’s r, 92 for MANOVA (two groups, five dependent variables), and 77 for multiple regression (three predictors). The final sample of 511 adolescents therefore exceeded these requirements, ensuring sufficient statistical power for the planned analyses.

Participants were divided into three age groups to allow comparisons across distinct age ranges commonly used in research on adolescence and emerging adulthood. Early (12–15 years) and middle adolescence (16–18 years) were defined based on developmental sub-stages widely adopted in the literature (Blum et al., 2014; Salmela-Aro, 2011). The 19–24 age group corresponds to the period often conceptualized as emerging adulthood (Salmela-Aro, 2011).

These older participants were included because they cohabited with younger residents within the same residential care institutions, sharing bedrooms and facilities, and following the same institutional rules and routines regardless of age. Interactions between older and younger residents are common, particularly while older await placement in units designed to support the transition toward autonomy.

The division between 12–15 and 16–18 age groups also aimed to ensure reasonable sized subgroups that reflect the most prevalent ages in residential care in Portugal and align with national educational cycles, which constitute meaningful age-related contexts for comparison.

### 2.2. Measures

#### 2.2.1. Strengths and Difficulties Questionnaire (SDQ, Goodman, 1997; Portuguese Version by Pechorro et al., 2011)

The SDQ is a 25-item self-report measure to evaluate socio-emotional issues through a three-point Likert scale. Items are organized into five subscales relating to emotional problems (e.g., “I am often unhappy, down-hearted or tearful”), behavioral problems (e.g., “I get very angry and often lose my temper”), hyperactivity/inattention difficulties (e.g., “I am restless, I cannot stay still for long”), peer relationship problems (e.g., “I am usually on my own. I generally play alone or keep to myself”), and prosocial behaviors (e.g., “I try to be nice to other people. I care about their feelings”) and a total difficulties score assesses the overall child’s mental health (Goodman, 1997). Original Cronbach’s alphas ranged from .65 to .85, while the Portuguese adaptation (Pechorro et al., 2011) showed values between .43 and .61. For the present study, reliability values obtained were: emotional problems α = .68, behavioral problems α = .57, hyperactivity/inattention difficulties α = .66, peer relationship problems α = .51, prosocial behaviors α = .78, and total scale α = .78.

#### 2.2.2. Short Dark Triad (SD3, Jones & Paulhus, 2014; Portuguese Version by Pechorro et al., 2018)

The Portuguese version assesses the DT traits with 21 items divided into three subscales: Machiavellianism (e.g., “Avoid direct conflict with others because they may be useful in the future”), narcissism (e.g., “I have been compared to famous people”), and psychopathy (e.g., “It’s true that I can be mean to others”). Participants rate their agreement on a 5-point Likert scale, with higher scores reflecting greater DT traits. Cronbach’s alpha in the original version was above .80; reliability values for the present study were: Machiavellianism α = .78, narcissism α = .58, and psychopathy α = .69.

An additional ad hoc questionnaire collected participants’ sociodemographic data, including age, sex, and length of stay in RCS.

### 2.3. Procedures

#### 2.3.1. Data Collection

Ethical approval for this study was obtained from the Ethics Committee of the University of Algarve (CEUAlg No. 110/2023). All procedures complied with the ethical standards outlined in the 1964 Declaration of Helsinki and its subsequent amendments. Permission to use the Portuguese version of the SD3 with youth from RCS was granted by the original authors of the instrument.

A convenience sampling strategy was used to recruit participants from 46 RCS across mainland Portugal and the Azores and Madeira archipelagos. Institutions were contacted via telephone and email, informed of this study’s objectives and procedures, and all agreed to participate. Data collection was conducted in person by the first author when possible. In cases where on-site administration was not feasible due to geographic constraints, questionnaires were sent by post and administered by institutional staff. Detailed written instructions and clarifications were provided during the authorization process and again upon delivery of the materials. The first author remained available throughout the data collection period to respond to any questions or concerns from staff or participants.

Inclusion criteria required participants to be at least 12 years old, fluent in Portuguese, and free from medical conditions that could impair their ability to participate. Adolescents identified by institutional staff as having cognitive impairments were excluded. All eligible participants were informed about this study’s aims, the voluntary nature of participation, and their right to withdraw at any point without consequence. Written informed consent was obtained from participants aged 16 and older. For those under 16, written consent was secured from a legal guardian or the institution’s technical director, in accordance with ethical guidelines.

Data were collected using anonymous, structured, self-report questionnaires administered within each institution. Additionally, institutional directors completed anonymous surveys providing information on organizational characteristics and religious affiliation.

Data collection took place between October 2023 and October 2024. Incomplete responses were excluded from the final dataset.

#### 2.3.2. Data Analysis

All descriptive and inferential statistical analyses were conducted using IBM SPSS Statistics for Windows software, Version 29 (IBM Corp, 2020). Statistical significance was set at *p* ≤ .05. Incomplete questionnaires were excluded from analyses, and missing data were handled through pairwise deletion.

To evaluate the study hypotheses, a range of analytical methods was employed, including correlation analysis, analysis of variance (ANOVA), multivariate analysis of variance (MANOVA), and multiple hierarchical regression (enter method). These analyses aimed to examine psychological adjustment as predicted by the Dark Triad traits and key demographic variables—namely, sex, age, and duration of institutionalization.

Because prior research indicates that boys and girls may differ in the expression and correlates of Dark Triad traits, sex-specific correlations were examined. Fisher’s z tests were then conducted to determine whether these correlations differed significantly between sexes (Cohen et al., 2003).

Age was categorized into three groups rather than treated as a continuous variable primarily for analytical and interpretative purposes. This grouping strategy aligns with prior research in developmental psychology, which frequently uses age brackets to represent key stages of maturation (e.g., early versus late adolescence). Given the cross-sectional design, the grouping was not intended to infer developmental trajectories but to allow comparisons between distinct segments of the sample and to identify potential differences in psychological profiles across age ranges.

Differences in psychological adjustment and the three dimensions of the SD3 across sex and age groups were analyzed using a multivariate analysis of variance (MANOVA), after confirming the assumptions of multivariate normality and homogeneity of variance-covariance matrices. Since SPSS Statistics does not directly assess multivariate normality, this assumption was verified using univariate Shapiro–Wilk tests (*p* ≥ .05 for all groups). No multivariate outliers were identified based on Mahalanobis distance. The assumption of no multicollinearity among the independent variables was examined by inspecting intercorrelations and Variance Inflation Factors (VIF) for the Dark Triad traits. Intercorrelations were below commonly accepted thresholds for concern, and all VIF values were within acceptable limits, indicating that multicollinearity was not problematic. With regard to assumptions related to the dependent variables, the absence of strong correlations among them supported the assumption of no multicollinearity at the outcome level. Homogeneity of variance-covariance matrices was assessed using Box’s M test, which yielded a statistically significant result, *M* = 30.95, *F*(15, 971225.707) = 2.041; *p* = .010.

Despite the significance of Box’s M, Pillai’s Trace was selected as the primary multivariate test statistic, given its robustness to violations of MANOVA assumptions, including heterogeneity of covariance matrices and deviations from multivariate normality (Olson, 1976; Tabachnick & Fidell, 2013). Pillai’s Trace is also preferred in cases of unequal group sizes or smaller sample due to its higher statistical power compared to Wilks’ Lambda.

When the MANOVA indicated statistically significant effects, follow-up univariate ANOVAs were conducted for each dependent variable, followed by Tukey’s HSD post hoc tests. A significance level of α = .05 was considered (Field, 2024).

## 3. Results

We began the analysis by examining associations between the Strengths and Difficulties Questionnaire (SDQ) total score, prosocial behavior, length of time in residential care, and participants’ age. Among females, older age was associated with fewer adjustment problems (*r* = −.26, *p* < .001), as was longer duration in institutional care (*r* = −.26, *p* < .001). For both sexes, age was positively correlated with prosocial behavior (females: *r* = .19, *p* < .01; males: *r* = .17, *p* < .01). Across the sample, higher levels of prosocial behavior were associated with fewer adjustment problems (*r* = −.18, *p* < .01).

Nest, we examined associations across all Dark Triad dimensions separately by sex (Table 1), and we provide age-group descriptives for all dimensions analyzed. As shown in Table 1, significant associations were observed across the personality traits, with boys scoring higher than girls on each dimension. Among girls, all correlations were significant except for those between narcissism and internalizing symptoms, externalizing behaviors, and SDQ total score. For boys, exceptions included the correlations between Machiavellianism and narcissism, and between the SDQ total score and prosocial behavior.

Weak negative correlations were found, for both sexes, between the SDQ total score and prosocial behavior, as well as between prosocial behavior and psychopathy. These findings indicate that higher levels of prosocial behavior are associated with fewer adjustment problems and lower levels of psychopathy.

Notably, psychopathy was the only dimension that showed significant associations across all outcome measures for both sexes.

Moderate positive correlations between Dark Triad dimensions were observed for both sexes. These findings indicate that the three Dark Triad traits are interrelated and share some common characteristics, and they further suggest that higher levels of these traits are associated with greater adjustment difficulties.

Among girls, correlations between Machiavellianism, prosocial behavior, and the SDQ total score indicate that higher levels of Machiavellianism are linked to increased psychological adjustment difficulties.

For both sexes, internalizing and externalizing behaviors were significantly associated with Machiavellianism and psychopathy, suggesting that these traits are strongly related to adolescents’ psychological adjustment.

To determine whether the correlations differed significantly between boys and girls, Fisher’s *z* tests were conducted for all pairs of variables. Most correlations did not differ by sex; however, three associations were significantly stronger among girls than boys: the correlations between externalizing symptoms and Machiavellianism (*z* = 3.70, *p* < .001), between SDQ total difficulties and Machiavellianism (*z* = 3.42, *p* = .001), and between externalizing symptoms and psychopathy (*z* = 2.00, *p* = .045). In contrast, the correlation between Machiavellianism and narcissism was significantly stronger among boys (*z* = –1.99, *p* = .047). These findings indicate that, although the overall pattern of associations was broadly similar across sexes, certain relations involving Machiavellianism and psychopathy were more pronounced in girls, whereas the link between Machiavellianism and narcissism was stronger in boys.

Table 2 presents the results of a multivariate analysis of variance (MANOVA) conducted to examine sex differences while controlling for age group.

The MANOVA revealed significant effects of sex and age group on psychological adjustment and Dark Triad traits [sex: Pillai’s Trace = 0.11, *F*(5, 504) = 12.17, *p* < .001, *η*^2^ = .11; age: Pillai’s Trace = 0.08, *F*(5, 504) = 8.54, *p* < .001; *η*^2^ = .08].

Follow-up univariate ANOVAs indicated significant sex differences for Machiavellianism, *F*(1, 509) = 8.11, *p* = .005, and psychopathy, *F*(1, 509) = 11.18, *p* < .001.

Pairwise comparisons using Tukey’s post hoc test revealed significant differences across age groups for all dependent variables except narcissism, suggesting that age-group differences are present for most socio-emotional and personality-related measures, with narcissism showing relative stability across the examined age range.

Regarding prosocial behavior, participants aged 12–15 (*M* = 6.93, *SD* = 2.32) scored lower than those aged 19–24 (*M* = 8.33, *SD* = 2.24, *p* < .001). For the SDQ total score, participants aged 12–15 (*M* = 16.50, *SD* = 6.47) and 16–18 (*M* = 15.46, *SD* = 5.87) scored higher than those aged 19–24 (*M* = 12.19, *SD* = 5.58), both *p* < .001, indicating fewer adjustment problems among the older age group.

Significant age-group differences were also observed for Machiavellianism, with participants aged 12–15 (*M* = 2.95, *SD* = 0.80) scoring higher than those aged 16–18 (*M* = 2.70, *SD* = 0.82), *p* = .004, and 19–24 (*M* = 2.62, *SD* = 0.88), *p* = .017. For psychopathy, participants aged 12–15 (*M* = 2.60, *SD* = 0.76) scored higher than those aged 19–24 (*M* = 2.13, *SD* = 0.64; *p* < .001, and *p* = .003, respectively).

Finally, a hierarchical multiple regression analysis was conducted to examine which variables were associated with psychological adjustment (Table 3).

In Step 1, sociodemographic variables were entered into the model. Both age (*β* = –.16, *t* = –3.33, *p* < .001) and sex (*β* = –.20, *t* = –5.08, *p* < .001) were significantly associated with psychological adjustment, with older participants and females reporting lower levels of adjustment difficulties.

In Step 2, the three Dark Triad traits were added, which significantly increased the explained variance. Psychopathy was positively associated with higher levels of adjustment difficulties (*β* = .47, *t* = 9.38, *p* < .001), whereas narcissism showed a small but significant negative association (*β* = –.12, *t* = –2.68, *p* = .008), suggesting a potential protective effect in this sample.

The final model was statistically significant, *F*(6, 504) = 27.956, *p* < .001, *R*^2^ = .25, indicating that both sociodemographic characteristics and Dark Triad traits were associated with psychological adjustment, with personality traits—particularly psychopathy—showing the strongest associations.

## 4. Discussion

The Dark Triad has been recognized as relevant for understanding delinquent behavior and the psychological and emotional factors underlying antisocial tendencies in adolescents. It also contributes to explaining variations in psychological adjustment, with adolescent self-reports of personality shown to be valid and reliable. However, research on the Dark Triad in individuals under the age of 18 remains limited (Pechorro et al., 2021), and to our knowledge, these traits have not been previously examined in adolescents residing in RCS.

The present study examined associations between Dark Triad traits and psychological adjustment in adolescents living in RCS, while also exploring sex and age differences in both domains. Consistent with prior literature, our findings support the conceptualization of Machiavellianism, narcissism, and psychopathy as distinct yet interrelated constructs in adolescence. The relatively large sample allowed for detailed analysis of age- and sex-based variations.

In line with our first hypothesis, younger adolescents exhibited higher levels of Machiavellianism and psychopathy, whereas older participants showed lower levels across all three traits. These patterns are consistent with previous research and conceptual frameworks suggesting that the expression of Dark Triad traits varies across adolescence (Klimstra et al., 2014, 2020). Although our study is cross-sectional and cannot infer developmental change, these age-group differences may reflect normative variation in personality traits during adolescence (Bonfá-Araujo et al., 2025; Schwaba et al., 2022). These findings support the view that adolescence is a critical developmental period for the emergence and consolidation of dark personality traits, emphasizing the importance of examining these traits within developmental and environmental contexts. These developmental patterns may also reflect the maturation of empathy and moral reasoning during adolescence (Hoffman, 2000; Sijtsema et al., 2019). Increases in perspective-taking and empathic concern are associated with the internalization of moral norms (Hoffman, 2000), which may mitigate socially aversive traits. Given that moral disengagement and psychopathy are still forming during this period, institutional environments could play a critical role in shaping their developmental trajectories.

Sex differences were observed, with males scoring higher in Machiavellianism and psychopathy, confirming our second hypothesis and aligning with prior research on gendered patterns of affective and behavioral expression (Furnham et al., 2013; Klimstra et al., 2020).

Muris et al. (2022) suggested that individuals high in Dark Triad traits—often linked to manipulative behaviors—may attempt to present themselves more favorably, potentially underreporting traits perceived as socially undesirable. Despite this tendency, the observed age and sex differences in self-reports align with prior research, reinforcing their validity.

Across internalizing and externalizing domains, females reported higher levels of both types of behaviors, alongside greater prosocial behavior and overall psychological difficulties. This pattern contrasts with earlier studies reporting higher externalizing behaviors among boys (Campos et al., 2019; Cotton et al., 2020) and partially supports our third hypothesis, highlighting the importance of accounting for sex-specific patterns in mental health assessment.

Regarding age, younger adolescents in our sample exhibited higher internalizing and externalizing problems, reduced prosocial behavior, and greater overall psychological maladjustment, whereas older participants showed higher prosocial behavior and fewer adjustment difficulties. These findings are consistent with prior studies indicating a decrease in both internalizing and externalizing problems with age (McWey et al., 2010; Tackett et al., 2014), supporting our fourth hypothesis. Some researchers have identified externalizing behavior disorders as the most predominant and challenging issues among children in alternative care (Keil & Price, 2006). Our findings corroborate the high rates of emotional and behavioral problems reported in child welfare populations (Keil & Price, 2006; Molano et al., 2024) and align with national data indicating that approximately one-quarter of youth entering RCS present with behavioral difficulties (Instituto da Segurança Social, 2024).

This study also examined associations between Dark Triad traits and psychological adjustment. Both sociodemographic variables and Dark Triad traits were associated with adjustment, with personality traits—particularly psychopathy—showing the strongest associations, supporting our fifth hypothesis. Higher levels of psychopathy were linked to increased tendencies toward behavioral difficulties and antisocial behavior (Pechorro et al., 2022), potentially reflecting characteristics such as limited concern for consequences and reduced fear of punishment (Lau & Marsee, 2013). While all three Dark Triad traits are associated with negative psychosocial outcomes, the literature suggests that, in adolescence, Machiavellianism and especially psychopathy are more robustly linked to maladaptive behaviors than narcissism (Muris et al., 2013). These findings are consistent with our results, which underscore psychopathy and Machiavellianism as particularly salient markers in the development of disruptive behaviors among youth (Zhao & Jin, 2023), warranting deeper exploration in this context.

Length of time in residential care was positively associated with adjustment in this sample, while some studies report negative effects of long-term institutionalization (Molano et al., 2024). These findings align with research suggesting that structured and secure residential care environments can provide stability and support for youths who have experienced early adversity. This association may also relate to processes of adaptation and adjustment following separation from family, consistent with perspectives from grief theory (Kübler-Ross & Kessler, 2005). Grief involves a complex emotional adaptation process encompassing physiological, cognitive, and behavioral components. Consequently, those who have spent less time in care may exhibit greater difficulty adjusting due to the ongoing grieving process.

Environmental and individual factors likely interact with Dark Triad traits. Adolescents in RCS often experience multiple adversities, including abuse, neglect, and separation, which may contribute to the observed associations between these traits and adjustment difficulties. Longitudinal evidence suggests bidirectional influences between personality traits and problem behaviors (Muris et al., 2017). In this context, higher levels of socially aversive traits may coincide with greater internalizing and externalizing difficulties, while exposure to such difficulties in institutional care may reinforce these traits. For youth struggling with emotional adaptation following placement, fostering acceptance of their situation is critical for achieving greater emotional stability. Additionally, age at the time of placement and the level of social integration are important factors, as adjustment within RCS may improve over time.

Consistent with prior research, psychopathy emerged as a particularly salient trait linked to behavioral maladjustment, with Machiavellianism also showing notable associations. Narcissism was less consistently related to maladaptive outcomes. These findings underscore the importance of considering the shared unique contributions of the Dark Triad traits to psychological adjustment, particularly in high-risk populations such as adolescents in residential care (Furnham et al., 2013; Muris et al., 2013).

Overall, our results highlight the relevance of the Dark Triad for underscoring socio-emotional functioning and behavioral adjustment in adolescents living in residential care. They emphasize the value of examining multiple traits simultaneously and considering both sociodemographic and environmental factors when interpreting associations with psychological adjustment.

### Limitations and Strengths

Although this study yielded important findings, several limitations should be acknowledged. First, this study relied on single-informant measures, which is a limitation particularly relevant to youth in RCS, who often have multiple caregivers. The reliance on self-reported data to assess Dark Triad traits may raise concerns, particularly given the manipulative tendencies associated with high levels of Machiavellianism. One might expect self-representation biases to influence responses; however, previous research, along with findings from the present study, suggests that self-reports on the Dark Triad can yield valid results. Future research should incorporate multi-informant assessments of the SDQ and SD3 to enhance the validity of findings. Multi-informant data may also help determine whether caregivers report SDQ scores within clinical ranges, as suggested in prior literature. Additionally, assessing social desirability bias could clarify potential distortions in adolescents’ self-reports.

Second, the cross-sectional design precludes causal inference. While Dark Triad traits have been linked to problematic behaviors in prior research, longitudinal studies are needed to clarify the directionality of these associations (Maier et al., 2023). Third, this study focused exclusively on subclinical levels of Dark Triad traits. Consequently, the findings cannot be generalized to clinical populations such as individuals diagnosed with narcissistic personality disorder or psychopathy. Nevertheless, given the continuum between normative personality traits and personality pathology, there is no theoretical basis to assume substantially different patterns of association in clinical populations. Future research should examine these associations in clinical samples and among youth in specialized settings such as residential care or therapeutic communities for addiction. Future works should also investigate differences in Dark Triad traits according to reasons for placement, given the significant role of adverse childhood experiences in shaping personality and psychological adjustment.

Fourth, although results seemed consistent with literature, the MANOVA including sex and age groups should be interpreted with caution, as the substantial discrepancy in group sizes—most notably the much smaller oldest age group—may compromise the statistical power and the stability of the multivariate estimates. This imbalance represents a methodological limitation of the current study.

Despite these limitations, this study has several notable strengths. These include the use of a national sampling frame, high response rates, and self-reports collected directly from a hard-to-reach and under-researched population. While the national scope of the sample is a significant strength, it is important to note that the structure and functioning of residential care and child welfare systems differ across countries. Thus, caution is warranted when generalizing findings beyond the Portuguese context.

## 5. Conclusions and Implications for Practice

This study examined Dark Triad traits in adolescents living in residential care and their association with psychological adjustment, offering new insights into this high-risk population. Both sociodemographic factors and Dark Triad traits—especially psychopathy—were significant predictors of adjustment. These findings underscore the need for early identification of youth at risk for mental health difficulties and the integration of psychological adjustment assessments into routine practice to guide individualized care planning. Evaluating Dark Triad traits may further support early detection and targeted interventions.

Effective support for youth in residential care requires routine psychological screening and timely access to individualized interventions. Improving the quality of parental support and strengthening preventive programs may also reduce the need for institutional placements (Giraldi et al., 2022). Staff working in RCS should receive specialized training to manage the personality-related challenges, and gender-sensitive interventions, mentoring, and structured group activities may enhance psychosocial well-being.

Because this study focuses on Portuguese RCS, generalizations should be made cautiously. Future research should examine how cultural, institutional and caregiving contexts interact with personality traits, and how factors such as trauma history, family contact, and attachment patterns may moderate psychosocial outcomes.

## Figures and Tables

**Table 1:** Descriptive statistics and correlations between Dark Triad traits and psychological adjustment by sex (*N*_girls_ = 279, *N*_boys_ = 232).

	1	2	3	4	5	6	7
1. Internalizing symptoms	-	.48 ***	.85 ***	−.03	.30 ***	.06	.34 ***
2. Externalizing symptoms	.31 ***	-	.87 ***	−.27 ***	.39 ***	.05	.50 ***
3. SDQ total score	.81 ***	.81 ***	-	−.18 **	.40 ***	.06	.49 ***
4. Prosocial behavior	−.01	−.29 ***	−.18 **	-	−.20 ***	.22 ***	−.23 ***
5. Machiavellianism	.18 **	.02	.12	−.10	-	.31 ***	.63 ***
6. Narcissism	.16 *	−.03	.08	.15 *	.49 ***	-	.33 ***
7. Psychopathy	.30 ***	.37 ***	.42 ***	−.22 ***	.56 ***	.44 ***	-
*M*_females_ (*SD*_females_)	8.62	7.73	16.35	7.79	2.71	3.03	2.39
	(3.65)	(3.85)	(6.46)	(2.13)	(0.79)	(0.63)	(0.76)
*M*_males_ (*SD*_males_)	7.13	7.45	14.59	6.87	2.92	3.09	2.62
	(3.59)	(3.61)	(5.82)	(2.42)	(0.86)	(0.67)	(0.74)
*M*_12–15_ (*SD*_12–15_)	8.08 (3.84)	8.44 (3.84)	16.50 (6.47)	6.93 (2.32)	2.95 (0.80)	3.03 (0.64)	2.60 (0.76)
*M*_16–18_ (*SD*_16–18_)	8.05 (3.58)	7.41 (3.48)	15.46 (5.87)	7.56 (2.32)	2.70 (0.82)	3.11 (0.66)	2.48 (0.75)
*M*_19–24_ (*SD*_19–24_)	7.09 (3.48)	5.10 (3.14)	12.19 (5.58)	8.33 (2.24)	2.62 (0.88)	2.98 (0.62)	2.13 (0.64)

Note. *r* values for girls on the upper-right section and boys score on the lower-left section; SDQ = Strengths and Difficulties Questionnaire. * *p* < .05; ** *p* < .01; *** *p* < .001.

**Table 2:** Multivariate comparison between sex and age group.

	*F*	Partial *η*^2^
**Control variable**		
Age	8.54 ***	.08
**Independent variable**		
Sex (females/males)	12.17 ***	.11
SDQ Total	10.34 ***	.02
Prosocial behavior	20.92 ***	.04
Machiavellianism	8.11 ***	.02
Narcissism	1.14	.00
Psychopathy	11.18 ***	.02

Note. SDQ = Strengths and Difficulties Questionnaire. *p* < .001 ***.

**Table 3:** Regression analysis of psychological adjustment (*N* = 511).

	Psychological Adjustment			
	∆*R*^2^	*F* Change	*β*	*t*
Step 1: Sociodemographic variables	.06	9.83 ***		
Age			−.16	−3.33 ***
Sex			−.20	−5.08 ***
Time in institution			−.05	−1.20
Step 2: SD3 dimensions	.20	43.61 ***		
Machiavellianism			.03	0.62
Narcissism			−.12	−2.68 *
Psychopathy			.47	9.38 ***

Note. SD3 = Short Dark Triad. * *p* < .05; *** *p* < .001.

## Data Availability

The data that support the findings of this study are available from the corresponding author upon reasonable request.

## Abbreviations

The following abbreviations are used in this manuscript:

DT	Dark Triad
RCS	Residential care settings
SDQ	Strengths and Difficulties Questionnaire
SD3	Short Dark Triad

## References

[B1-behavsci-16-00037] Almas A. N., Papp L. J., Woodbury M. R., Nelson C. A., Zeanah C. H., Fox N. A. (2020). The impact of caregiving disruptions of previously institutionalized children on multiple outcomes in late childhood. Child Development.

[B2-behavsci-16-00037] Bandura A. (1977). Social learning theory.

[B3-behavsci-16-00037] Barone L., Dellagiulia A., Lionetti F. (2016). When the primary caregiver is missing: Investigating proximal and distal variables involved in institutionalised children’s adjustment. Child Abuse Review.

[B4-behavsci-16-00037] Blum R. W., Astone N. M., Decker M. R., Mouli V. C. (2014). A conceptual framework for early adolescence: A platform for research. International Journal of Adolescent Medicine and Health.

[B5-behavsci-16-00037] Bonfá-Araujo B., Magarotto Machado G., Caurin N. B., Lima-Costa A. R. (2025). Could the younger be darker? A cross-cectional study on the Dark Triad levels across the lifespan. Deviant Behavior.

[B6-behavsci-16-00037] Campos J., Barbosa-Ducharne M., Dias P., Rodrigues S., Martins A. C., Leal M. (2019). Emotional and behavioral problems and psychosocial skills in adolescents in residential care. Adolescent Social Work Journal.

[B7-behavsci-16-00037] Chiorri C., Garofalo C., Velotti P. (2019). Does the Dark Triad manifest similarly in men and women? Measurement invariance of the Dirty Dozen across sex. Current Psychology.

[B8-behavsci-16-00037] Cohen J., Cohen P., West S. G., Aiken L. S. (2003). Applied multiple regression/correlation analysis for the behavioral sciences.

[B9-behavsci-16-00037] Costa M., Mota C. P., Matos P. M. (2022). Predictors of psychosocial adjustment in adolescents in residential care: A systematic review. Child Care in Practice.

[B10-behavsci-16-00037] Cotton S. M., Rice S., Moeller-Saxone K., Magnus A., Harvey C., Mihalopoulos C., Humphreys C., Murray L., Halperin S., McGorry P. D., Herrman H. (2020). Sex differences in psychological distress, behavioural and emotional problems, and substance use in young people in out-of-home care. Child & Family Social Work.

[B11-behavsci-16-00037] Csathó A., Birkás B. (2018). Early-life stressors, personality development, and fast life strategies: An evolutionary perspective on malevolent personality features. Frontiers in Psychology.

[B12-behavsci-16-00037] Diller S. J., Czibor A., Szabó Z. P., Restás P., Jonas E., Frey D. (2021). The positive connection between dark triad traits and leadership levels in self- and other-ratings. Leadership, Education, Personality: An Interdisciplinary Journal.

[B13-behavsci-16-00037] Faul F., Erdfelder E., Lang A.-G., Buchner A. (2007). G*Power 3: A flexible statistical power analysis program for the social, behavioral, and biomedical sciences. Behavior Research Methods.

[B14-behavsci-16-00037] Fernández-del-Río E., Ramos-Villagrasa P. J., Barrada J. R. (2020). Bad guys perform better? The incremental predictive validity of the Dark Tetrad over Big Five and Honesty-Humility. Personality and Individual Differences.

[B15-behavsci-16-00037] Field A. (2024). Discovering statistics using IBM SPSS statistics.

[B16-behavsci-16-00037] Furnham A., Richards S. C., Paulhus D. L. (2013). The Dark Triad of personality: A 10-year review. Social and Personality Psychology Compass.

[B17-behavsci-16-00037] Galán M., Pineda D., Rico-Bordera P., Piqueras J. A., Muris P. (2025). Dark childhood, dark personality: Relations between experiences of child abuse and dark tetrad traits. Personality and Individual Differences.

[B18-behavsci-16-00037] Giraldi M., Mitchell F., Porter R. B., Reed D., Jans V., McIver L., Manole M., McTier A. (2022). Residential care as an alternative care option: A review of literature within a global context. Child & Family Social Work.

[B19-behavsci-16-00037] Goodman R. (1997). The strengths and difficulties questionnaire: A research note. Journal of Child Psychology and Psychiatry.

[B20-behavsci-16-00037] Götz F. M., Bleidorn W., Rentfrow P. J. (2020). Age differences in Machiavellianism across the life span: Evidence from a large-scale cross-sectional study. Journal of Personality.

[B21-behavsci-16-00037] Hair J. F., Black W. C., Babin B. J., Anderson R. E. (2010). Multivariate data analysis.

[B22-behavsci-16-00037] Hayden C. (2010). Offending behaviour in care: Is children’s residential care a ‘criminogenic’ environment?. Child & Family Social Work.

[B23-behavsci-16-00037] Hoffman M. L. (2000). Empathy and moral development.

[B24-behavsci-16-00037] Hu Y., Lan X. (2022). A comprehensive and person-centered view of the association between the Dark Triad and youth mental health. Frontiers in Psychiatry.

[B25-behavsci-16-00037] IBM Corp (2020). IBM SPSS statistics for windows *(Version 29.0)*.

[B26-behavsci-16-00037] Instituto da Segurança Social I. P. (2024). CASA 2023: Relatório de caracterização anual da situação de acolhimento das crianças e jovens.

[B27-behavsci-16-00037] Jonason P. K., Burtăverde V., Buss D. M. (2023). The dark triad traits and mating psychology. The Oxford handbook of human mating.

[B28-behavsci-16-00037] Jonason P. K., Davis M. D. (2018). A gender role view of the Dark Triad traits. Personality and Individual Differences.

[B29-behavsci-16-00037] Jonason P. K., Duineveld J. J., Middleton J. P. (2015). Pathology, pseudopathology, and the Dark Triad of personality. Personality and Individual Differences.

[B30-behavsci-16-00037] Jonason P. K., Foster J. D., Egorova M. S., Parshikova O., Csathó Á., Oshio A., Gouveia V. V. (2017). The Dark Triad traits from a life history perspective in six countries. Frontiers in Psychology.

[B31-behavsci-16-00037] Jonason P. K., Tost J. (2010). I just cannot control myself: The Dark Triad and self-control. Personality and Individual Differences.

[B32-behavsci-16-00037] Jones D. N., Paulhus D. L. (2014). Introducing the short dark triad (SD3). Assessment.

[B33-behavsci-16-00037] Keil V., Price J. M. (2006). Externalizing behavior disorders in child welfare settings: Definition, prevalence, and implications for assessment and treatment. Children and Youth Services Review.

[B34-behavsci-16-00037] Klimstra T. A., Jeronimus B. F., Sijtsema J. J., Denissen J. J. A. (2020). The unfolding dark side: Age trends in dark personality features. Journal of Research in Personality.

[B35-behavsci-16-00037] Klimstra T. A., Sijtsema J. J., Henrichs J., Cima M. (2014). The Dark Triad of personality in adolescence: Psychometric properties of a concise measure and associations with adolescent adjustment from a multi-informant perspective. Journal of Research in Personality.

[B36-behavsci-16-00037] Koehn M. A., Okan C., Jonason P. K. (2019). A primer on the Dark Triad traits. Australian Journal of Psychology.

[B37-behavsci-16-00037] Kübler-Ross E., Kessler D. (2005). On grief and grieving: Finding the meaning of grief through the five stages of loss.

[B38-behavsci-16-00037] Lau K. S. L., Marsee M. A. (2013). Exploring narcissism, psychopathy, and Machiavellianism in youth: Examination of associations with antisocial behavior and aggression. Journal of Child and Family Studies.

[B39-behavsci-16-00037] Law 142/2015 (2015). The law for protection of children and youth at risk *[Lei de Proteção de Crianças e Jovens em Perigo]*.

[B40-behavsci-16-00037] Li J., Liu C., Albertella L., Rotaru K., Li K., Zhou Y., Wei X., Yuan S., Liu X., Ren L. (2024). Network analysis of the association between Dark Triad traits and depression symptoms in university students. Personality and Individual Differences.

[B41-behavsci-16-00037] Magalhães E., Camilo C. (2023). Maltreatment history and internalizing and externalizing symptoms in out-of-home care: A three-level meta-analysis. European Journal of Psychology Applied to Legal Context.

[B42-behavsci-16-00037] Maier C., Thatcher J. B., Grover V., Dwivedi Y. K. (2023). Cross-sectional research: A critical perspective, use cases, and recommendations for IS research. International Journal of Information Management.

[B43-behavsci-16-00037] Mayorga-Sierra É., Novo M., Fariña F., Seijo D. (2020). Needs analysis for the personal, social, and psychological adjustment of adolescents at risk of delinquency and juvenile offenders. Anales de Psicología.

[B44-behavsci-16-00037] McCrae R. R., Costa P. T. (1987). Validation of the five-factor model of personality across instruments and observers. Journal of Personality and Social Psychology.

[B45-behavsci-16-00037] McGuire A., Cho B., Huffhines L., Gusler S., Brown S., Jackson Y. (2018). The relation between dimensions of maltreatment, placement instability, and mental health among youth in foster care. Child Abuse & Neglect.

[B46-behavsci-16-00037] McWey L. M., Cui M., Pazdera A. L. (2010). Changes in externalizing and internalizing problems of adolescents in foster care. Journal of Marriage and Family.

[B47-behavsci-16-00037] Molano N., Espinosa E., León E. (2024). Psychological adjustment of children and adolescents in residential care with Collaborating Families: A multi-informant assessment. Anales de Psicología.

[B48-behavsci-16-00037] Moreno-Manso J. M., García-Baamonde M. E., Guerrero-Barona E., Godoy-Merino M. J., Guerrero-Molina M., Barbosa-Torres C. (2023). Externalizing and internalizing symptoms and coping strategies in young victims of abuse. Current Psychology.

[B49-behavsci-16-00037] Muris P., Jeurissen A., Rooswinkel M., Meesters C. (2022). Good traits, bad traits, and ‘ugly’ behavior: Relations between the Dark Triad, honesty-humility, other HEXACO personality traits, and externalizing problems in adolescents. Journal of Child and Family Studies.

[B50-behavsci-16-00037] Muris P., Meesters C., Timmermans A. (2013). Some youths have a gloomy side: Correlates of the Dark Triad personality traits in non-clinical adolescents. Child Psychiatry & Human Development.

[B51-behavsci-16-00037] Muris P., Merckelbach H., Otgaar H., Meijer E. (2017). The malevolent side of human nature. Perspectives on Psychological Science.

[B52-behavsci-16-00037] Olson C. L. (1976). On choosing a test statistic in multivariate analysis of variance. Psychological Bulletin.

[B53-behavsci-16-00037] Paknejad N., Mazandarani A. A. (2024). Psychopathy, Machiavellianism, and sleep patterns as predictors of adolescent depression. Current Psychology.

[B54-behavsci-16-00037] Paulhus D. L., Williams K. M. (2002). The dark triad of personality: Narcissism, Machiavellianism, and psychopathy. Journal of Research in Personality.

[B55-behavsci-16-00037] Pechorro P., Caramelo V., Oliveira J. P., Nunes C., Curtis S. R., Jones D. N. (2018). The Short Dark Triad (SD3): Adaptation and psychometrics among at-risk male and female youths. Deviant Behavior.

[B56-behavsci-16-00037] Pechorro P., Curtis S., DeLisi M., Maroco J., Nunes C. (2022). Dark Triad psychopathy outperforms self-control in predicting antisocial outcomes: A structural equation modeling approach. European Journal of Investigation in Health, *Psychology and Education*.

[B57-behavsci-16-00037] Pechorro P., Jonason P. K., Raposo V., Maroco J. (2021). Dirty Dozen: A concise measure of dark triad traits among at-risk youths. Current Psychology.

[B58-behavsci-16-00037] Pechorro P., Poiares C., Vieira R. (2011). Propriedades psicométricas do Questionário de Capacidades e de Dificuldades na versão portuguesa de auto-resposta/Psychometric properties of the Portuguese self-report version of the Strengths and Difficulties Questionnaire. Journal of Consultation-Liaison Psychiatry.

[B59-behavsci-16-00037] Penado Abilleira M., Rodicio-García M.-L., Ríos-de-Deus M.-P., Alonso del Hierro T. (2024). Adaptation of the Short Dark Triad (SD3) to Spanish adolescents. European Journal of Investigation in Health, *Psychology and Education*.

[B60-behavsci-16-00037] Rutter M. (2000). Children in substitute care: Some conceptual considerations and research implications. Children and Youth Services Review.

[B61-behavsci-16-00037] Salmela-Aro K., Brown B. B., Prinstein M. J. (2011). Stages of adolescence. Encyclopedia of adolescence.

[B62-behavsci-16-00037] Schwaba T., Bleidorn W., Hopwood C. J., Manuck S. B., Wright A. G. C. (2022). Refining the maturity principle of personality development by examining facets, close others, and comaturation. Journal of Personality and Social Psychology.

[B63-behavsci-16-00037] Shen K. (2023). The dark triad and depressive symptoms among Chinese adolescents: Moderated mediation models of age and emotion regulation strategies. Current Psychology.

[B64-behavsci-16-00037] Shiner R. L., Allen T. A., Masten A. S. (2017). Adversity in adolescence predicts personality trait change from childhood to adulthood. Journal of Research in Personality.

[B65-behavsci-16-00037] Sijtsema J. J., Garofalo C., Jansen K., Klimstra T. A. (2019). Disengaging from evil: Longitudinal associations between the Dark Triad, moral disengagement, and antisocial behavior in adolescence. Journal of Abnormal Child Psychology.

[B66-behavsci-16-00037] Simão A., dos Santos R. A. M., Brás M., Nunes C. (2025). Determinants of psychological adjustment of institutionalized adolescents: A systematic review. Child & Youth Care Forum.

[B67-behavsci-16-00037] Stelmokienė A., Vadvilavičius T. (2022). Can Dark Triad traits in leaders be associated with positive outcomes of transformational leadership: Cultural differences. Psihologijske Teme.

[B68-behavsci-16-00037] Szabó Z. P., Orosz N. Z., Gulyás R., Láng A. (2024). The associations of peer-rated popularity and likeability with Dark Triad personality traits in adolescent groups. Europe’s Journal of Psychology.

[B69-behavsci-16-00037] Tabachnick B. G., Fidell L. S. (2013). Using multivariate statistics.

[B70-behavsci-16-00037] Tackett J. L., Herzhoff K., Reardon K. W., De Clercq B., Sharp C. (2014). The externalizing spectrum in youth: Incorporating personality pathology. Journal of Adolescence.

[B71-behavsci-16-00037] Wang C., Guo J., Zhou X., Shen Y., You J. (2023). The Dark Triad traits and suicidal ideation in Chinese adolescents: Mediation by social alienation. Journal of Research in Personality.

[B72-behavsci-16-00037] Zhao B., Jin C. (2023). Mechanisms of driving adolescents’ adjustment in behavior: A developmental systems approach. Youth & Society.

